# *Candida albicans* increases the pathogenicity of *Staphylococcus aureus* during polymicrobial infection of *Galleria mellonella* larvae

**DOI:** 10.1099/mic.0.000892

**Published:** 2020-02-18

**Authors:** Gerard Sheehan, Laura Tully, Kevin A. Kavanagh

**Affiliations:** ^1^​ SSPC Pharma Research Centre, Department of Biology, Maynooth University, Maynooth, Co. Kildare, Ireland; ^2^​ Institute of Microbiology and Infection, School of Biosciences, University of Birmingham, Edgbaston, Birmingham, B15 2TT, UK

**Keywords:** *Galleria*, infection, immunity, mini-model, *Candida*, *Staphylococcus*, co-infection

## Abstract

This study detailed the responses of *Galleria mellonella* larvae to disseminated infection caused by co-infection with *Candida albicans* and *Staphylococcus aureus *. Doses of *C. albicans* (1×10^5^ larva^−1^) and *S. aureus* (1×10^4^ larva^−1^) were non-lethal in mono-infection but when combined significantly (*P*<0.05) reduced larval survival at 24, 48 and 72 h relative to larvae receiving *S. aureus* (2×10^4^ larva^−1^) alone. Co-infected larvae displayed a significantly higher density of *S. aureus* larva^−1^ compared to larvae infected solely with *S. aureus *. Co-infection resulted in dissemination throughout the host and the appearance of large nodules. Co-infection of larvae with *C. albicans* and *S. aureus* (2×10^4^ larva^−1^) resulted in an increase in the density of circulating haemocytes compared to that in larvae infected with only *S. aureus *. Proteomic analysis of co-infected larval haemolymph revealed increased abundance of proteins associated with immune responses to bacterial and fungal infection such as cecropin-A (+45.4-fold), recognition proteins [e.g. peptidoglycan-recognition protein LB (+14-fold)] and proteins associated with nodule formation [e.g. Hdd11 (+33.3-fold)]. A range of proteins were also decreased in abundance following co-infection, including apolipophorin (−62.4-fold), alpha-esterase 45 (−7.7-fold) and serine proteinase (−6.2-fold). Co-infection of larvae resulted in enhanced proliferation of *S. aureus* compared to mono-infection and an immune response showing many similarities to the innate immune response of mammals to infection. The utility of *G. mellonella* larvae for studying polymicrobial infection is highlighted.

## Introduction

The human microbiome consists of communities of bacteria, archaea, viruses and fungi that, through their interactions, have both positive and negative effects on each other and on their host [1]. In times of dysbiosis, polymicrobial infections can lead to aggressive forms of diseases that are usually difficult to treat. Co-colonization by *Aspergillus fumigatus* and *Pseudomonas aeruginosa* in the cystic fibrosis lung has poorer clinical outcomes compared to colonization by *P. aeruginosa* alone [2]. A large proportion of infections (30–60 %) caused by *Candida albicans* are polymicrobial and *Staphylococcus aureus* is one of the most common microbes isolated during these infections [3–5]. Infections caused by *C. albicans* affect large numbers of patients and a recent study found that in chemotherapy-immunosuppressed mice *C. albicans* can influence the resident bacterial community and contribute to mucosal bacterial dysbiosis, facilitating enhanced invasion by *Candida* [6]. In recent years, advances in sequencing technologies have demonstrated that *Candida* rarely exists in isolation but rather as a heterogeneous biofilm with other fungal and bacterial species [7]. These types of infections in humans include catheter infections, burn wound infections, septicaemia, ventilator-associated pneumonia, keratitis, denture stomatitis, peritonitis, cystic fibrosis and urinary tract infections [1, 8]. The interactions between *S. aureus* and *C. albicans* are synergistic and result in increased mortality in animal models, which is associated with enhanced invasion, biofilm formation, exacerbated inflammatory responses and intrinsic resistance to antimicrobial chemotherapy [8–19]. Proteomic analysis of the interactions between *S. aureus* and *C. albicans* has demonstrated an increased abundance of proteins associated with stress and growth responses, and metabolism in the early stages of the interaction, although *S. aureus* had little effect on the proteome of *C. albicans* hyphae [20].

*C. albicans* virulence includes polymorphism and phenotypic switching, production of toxins (e.g. candidalysin), invasins (e.g. Als3p), biofilm formation and metabolic adaption [21, 22]. The virulence of *S. aureus* is multifaceted and allows the bacterium to interact with the host (fibronectin-binding proteins A and B), evade the immune response (chemotaxis inhibitory protein of staphylococci) and induce tissue damage (alpha toxin) [23–27]. During co-infection *S. aureus* can bind to Als3p in the *C. albicans* cell wall and this facilitates disseminated infection in an oral co-colonization model [8]. Interestingly, interactions between *C. albicans* and *S. aureus* in mixed species biofilm result in alterations in the proteome, resulting in increased abundance of proteins associated with resistance to oxidative stress and virulence [28]. To date, these pathogens have mostly been studied in isolation in higher vertebrates and there is a lack of a system that is compatible with the 3R policies and enables the generation of rapid results and allows the screening of multiple interactions in a short space of time.

Larvae of the greater wax moth *Galleria mellonella* are a widely used model to study the virulence of microbial pathogens and to assess the efficacy of antimicrobial agents due to the many advantages associated with their use (e.g. ease of use, cost-effectiveness, lack of legal and ethical restrictions) [29–33]. More recently, larvae have been utilized to study the virulence of bacterial (e.g. *S. aureus* [34], *Streptococcus pneumoniae* [35] and *Enterococcus faecalis* [36, 37]) and fungal (e.g. *C. albicans* [38, 39], *A. fumigatus* [40] and *Cryptococcus neoformans* [41]) pathogens as well as their interactions with the cellular and humoral immune response of larvae. Larvae were also used to assess the interaction between *S. aureus* and *C. albicans* and show how co-infection reduced the efficacy of miconazole [14]. The aim of this work was to characterize the development of, and the host immune response to, mixed microbial co-infection in *G. mellonella* larvae in order to develop a simple but effective system to characterize polymicrobial infections *in vivo*.

## Methods

### Strains and culture conditions

*C. albicans* MEN (a kind gift from Professor D. Kerridge, Cambridge, UK) was cultured in YEPD broth [2 % (w/v) glucose, 2 % (w/v) bactopeptone (Difco Laboratories), 1 % (w/v) yeast extract (Oxoid Ltd, Basingstoke, UK)]. *S. aureus* (clinical isolate) was cultured in nutrient broth (Oxoid). Cultures were grown overnight at 37 °C and 200 r.p.m. to the early stationary phase as described elsewhere [34]. Cells were harvested by centrifugation (2000 x ***g***) and cell pellets were washed three times with phosphate-buffered saline (PBS) prior to injection into larvae. Stocks of *C. albicans* and *S. aureus* were maintained on YEPD agar plates as above but supplemented with 2 % (w/v) agar] or nutrient agar plates, respectively.

### Larval culture and inoculation

Sixth instar larvae of the greater wax moth *G. mellonella* (Livefoods Direct Ltd, UK) were stored in the dark at 15 °C and maintained in wood chippings [38]. Larvae weighing 0.22±0.03 g were selected and used within 2 weeks of receipt. Ten healthy larvae per treatment and controls were placed in sterile 9 cm Petri dishes lined with Whatman filter paper and containing some wood shavings. Larvae were inoculated with *S. aureus* (enumerated using a spectrophotometer) through the last left pro-leg into the haemocoel with a Myjector U-100 insulin syringe (Terumo Europe NV, Belgium). For co-infection, larvae were administrated 20 µl of a solution containing *C. albicans* (enumerated using a haemocytometer) and *S. aureus* at the inoculum stated. Larvae were acclimatized to 37 °C for 1 h prior to all experiments and incubated at 37 °C for all studies. All experiments were performed independently on three separate occasions.

### Determination of proliferation of *S. aureus* in *G. mellonella* larvae

Larvae were infected with *C. albicans* or *S aureus* or co-infected with *C. albicans* and *S aureus *, and *S. aureus* proliferation larva^−1^ was assessed over 48 h. Infected larvae (*n*=3) were homogenized using a pestle and mortar with 3 ml of PBS, serially diluted and plated onto nutrient agar (* S. aureus *) plates supplemented with 0.1 g (v/v) amphotericin B and incubated at 37 °C for 24 h. The bacterial load was calculated as the *S. aureus* colony-forming units (c.f.u.) per larva and was based on the number of colonies that grew at specific dilutions. This protocol is described in detail elsewhere [34, 42].

### Cryo-imaging of co-infected *G. mellonella* larvae

*G. mellonella* larvae were co-infected with *C. albicans* (1×10^5^ larva^−1^) and *S. aureus* (2×10^4^ larva^−1^) and incubated at 37 °C for 0, 6, 24 and 48 h. Larvae (*n*=3) were embedded in Cryo-Imaging Embedding Compound (Bioinvision), flash-frozen in liquid nitrogen and mounted on a stage for sectioning [34, 40]. Sectioning and imaging was carried out every 10 µm using a Cryoviz (Bioinvision, Inc., Cleveland, OH, USA) cryo-imaging system.

### Determination of haemocyte density in *G. mellonella* larvae

Changes in haemocyte density was assessed by bleeding 40 µl of haemolymph from *G. mellonella* larvae (*n*=5) into a microcentrifuge tube on ice to prevent melanization. Haemolymph was diluted in 0.37 % (v/v) mercaptoethanol-supplemented PBS and cell density was determined using a haemocytometer. Haemocyte density was expressed as haemocyte density per larva. Experiments were performed on three independent occasions and the means±se were determined.

### Quantitative proteomics of larval haemolymph

Quantitative proteomics was conducted on haemocyte-free haemolymph from larvae (*n*=10 with four biological replicates) at 0, 6 and 24 h post-co-infection [*C. albicans* (1×10^5^ larva^−1^), *S. aureus* (2×10^4^ larva^−1^)]. Protein (75 µg) was prepared according to established protocols [38]. Protein identification from the tandem mass spectrometry (MS/MS) data was performed using the Andromeda search engine in MaxQuant (version 1.2.2.5; http://maxquant.org/) to correlate the data against a six-frame translation of the EST contigs for *G. mellonella* [43, 44] or the proteome for *C. albicans* or *S. aureus* (Uniprot).

Results processing, statistical analyses and graphics generation were conducted using Perseus v 1.5.5.3. Label-free quantification (LFQ) intensities were log_2_-transformed and analysis of variance (ANOVA) of significance and *t*-tests between the haemolymph proteomes of 0, 6 and 24 h *S *. *aureus *-treated larvae were performed using a *P*-value of 0.05 and significance was determined using false discovery rate (FDR) correction (Benjamini–Hochberg). The (FDR was set at 1 % for both peptides and proteins. Proteins that had non-existent values (indicative of absence or very low abundance in a sample) were also used in statistical analysis of the total differentially expressed group following imputation of the zero values using a number close to the lowest value of the range of proteins plus or minus the standard deviation. After data imputation these proteins were included in subsequent statistical analysis.

### Statistical analysis

All experiments were performed on three independent occasions and the results are expressed as the mean±se. All of the statistical analysis listed was performed using GraphPad Prism v 6.00 (one-way ANOVA; survival, haemocyte density, c.f.u.). Differences were considered significant at *P*<0.05.

### Data availability

The mass spectrometry (MS) proteomics data and MaxQuant search output files have been deposited to the ProteomeXchange Consortium [45] via the PRIDE partner repository with the dataset identifier PXD014273.

## Results

### Response of *G. mellonella* larvae to co-infection by *C. albicans* and *S. aureus*

**Fig. 1.**
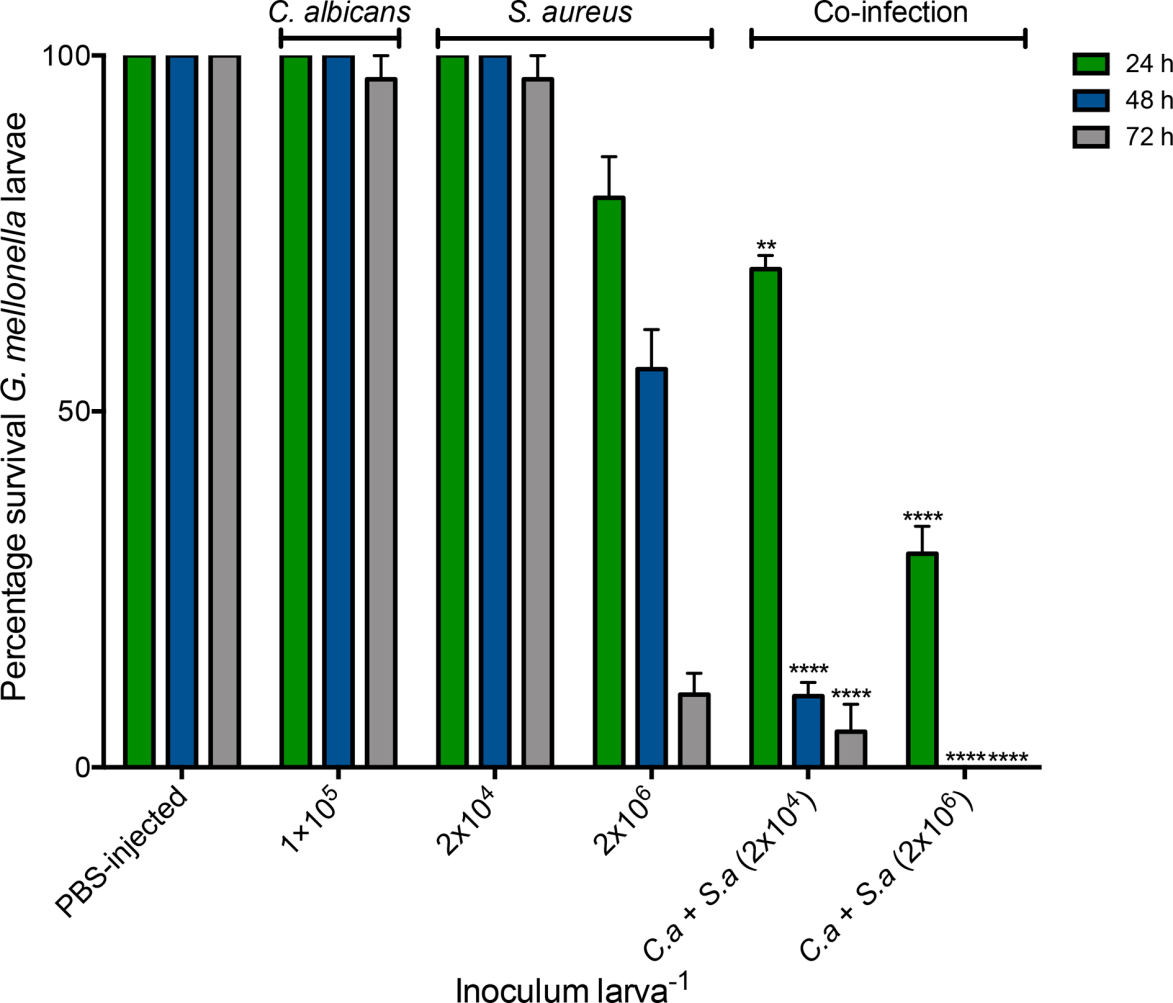
Survival of *G. mellonella* larvae following mono- and co- infection with *C. albicans* and *S. aureus *. Larvae were infected with 20 µl of *C. albicans* or *S. aureus* or co-infected (1×10^5^ *C. albicans* with varying concentrations of *S. aureus *) and survival was assessed over 72 h at 37 °C. Statistical significance was determined by comparing co-infected larvae to the relevant *S. aureus* mono-infected larvae (**, *P*<0.01; ****, *P*<0.0001). Larvae were also injected with 20 µl PBS (PBS-injected). All values are the mean±se of three independent experiments.

Infection of larvae with an inoculum of 1×10^5^ *C. albicans* larva^−1^ resulted in no significant decrease in survival over 72 h as compared to non-infected controls. Infection of larvae with *S. aureus* (2×10^6^ larva^−1^) resulted in larval survival of 80±10 % at 24 h, 55.93±9.62 % at 48 h and 10±5.15 % at 72 h post-infection (Fig. 1﻿﻿﻿). Co-infection of larvae with *C. albicans* (1×10^5^ larva^−1^) and *S. aureus* (2×10^4^ larva^−1^) resulted in a significant decrease in survival [24 h, 70±3.33 % (*P*<0.01); 48 h, 10±3.33 % (*P*<0.0001); 72 h, 5±6.66 % (*P*<0.0001)] compared to larvae that received *S. aureus* (2×10^4^ larva^−1^) alone at 24, 48 (100±0 %) and 72 h (96.66±5.77 %). Co-infection of *G. mellonella* larvae with *C. albicans* (1×10^5^ larva^−1^) and *S. aureus* (2×10^6^ larva^−1^) reduced survival to 30±6.66 % (*P*<0.0001) at 24 h and 0±0 % (*P*<0.0001) at 48 and 72 h post-infection, relative to mono-infection with *S. aureus* (2×10^6^ larva^−1^) (Fig. 1).

### Dissemination of infection throughout the host

**Fig. 2.**
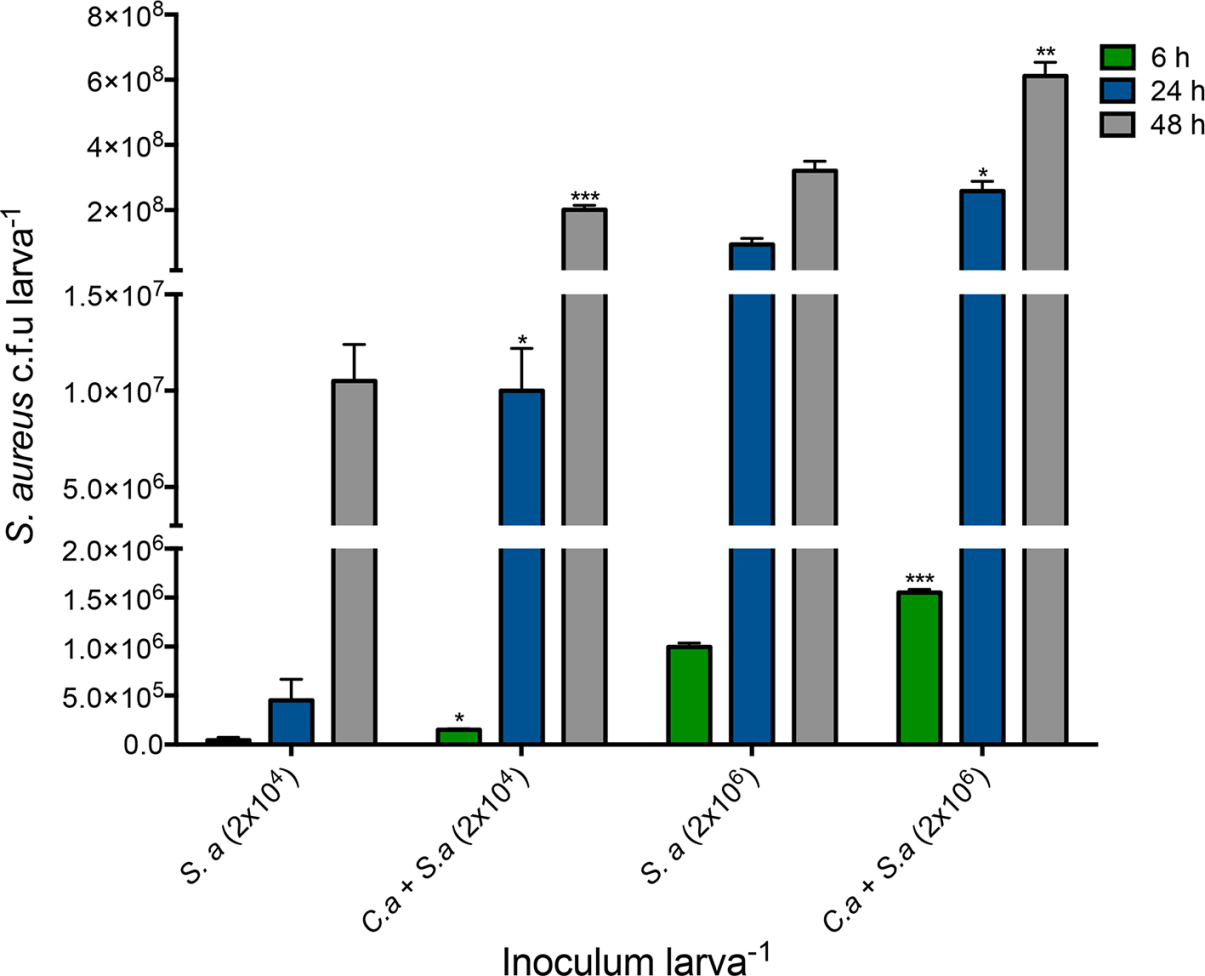
*
S. aureus* c.f.u. larva^−1^ obtained from *G. mellonella* larvae infected with *S. aureus* cells (2×10^6^ and 2×10^6^ larva^−1^) or co-infected larvae over 48 h at 37 °C. Co-infection with *C. albicans* and *S. aureus* (*C. a+S. a*) results in an increase in bacterial load in larvae from 6 to 48 h as determined by plating on nutrient agar plates. Statistical analysis was carried out by comparing co-infected larvae (*C. a+S. a*) to larvae infected solely with *S. aureus* (*S. a*) at their respective timepoints, (*, *P*<0.05; **, *P*<0.01). All values are the mean±se of three independent experiments.

The microbial burden of co-infected larvae was assessed by measuring *S. aureus* c.f.u larva^−1^ in co-infected [*C. albicans* (1×10^5^ larva^−1^) with *S. aureus* (2×10^4^ or 2×10^6^ larva^−1^)] larvae and comparing these to larvae infected solely with *S. aureus* (2×10^4^ or 2×10^6^ larva^−1^). Co-infected larvae displayed a significantly higher *S. aureus* c.f.u larva^−1^ at 6 (1.51±0.17×10^5^ c.f.u. larva^−1^, *P*<0.05), 24 (1.01±0.38×10^7^ c.f.u. larva^−1^, *P*<0.05) and 48 h (2.01±0.23×10^8^ c.f.u larva^−1^, *P*<0.001) compared to larvae infected with *S. aureus* (2×10^4^ larva^−1^) alone at 6 (4.49±0.5×10^4^ c.f.u larva^−1^), 24 (4.51±0.37×10^5^ c.f.u larva^−1^) and 48 h (1.05±0.33×10^7^ c.f.u larva^−1^). There was a significant increase in *S. aureus* c.f.u. at 6 (1.55±0.59×10^6^ c.f.u larva^−1^, *P*<0.001), 24 (2.58±0.52×10^8^ c.f.u larva^−1^, *P*<0.05) and 48 h (6.12±0.73×10^8^, *P*<0.01) post-infection in co-infected larvae compared to those infected solely with *S. aureus* (2×10^6^ larva^−1^) at 6 (9.96±0.68×10^5^ c.f.u. larva^−1^), 24 (9.41±0.34×10^7^ c.f.u. larva^−1^) and 48 h (3.21±0.51×10^8^ c.f.u. larva^−1^) (Fig. 2﻿).

**Fig. 3.**
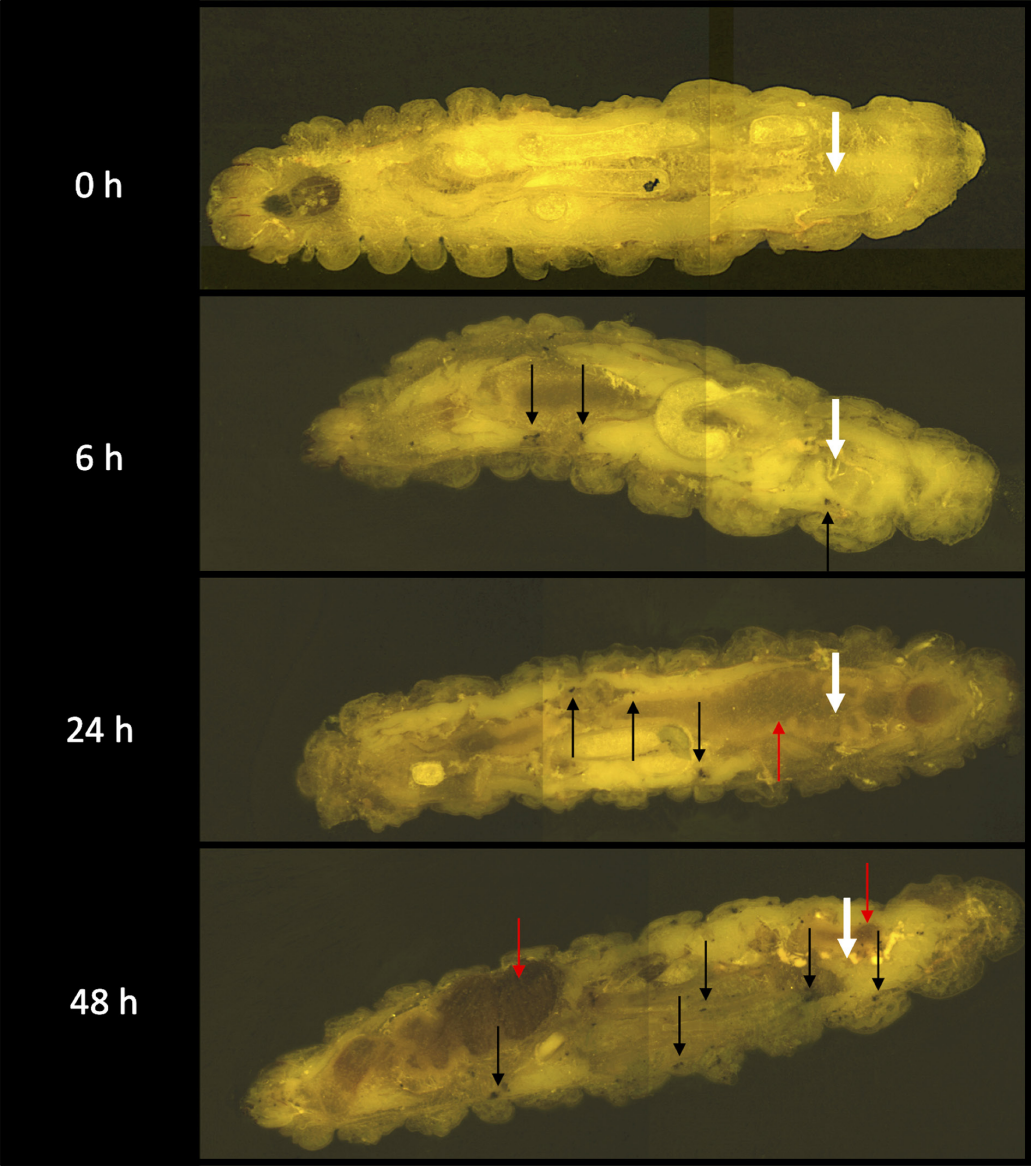
CryoViz-imaging of *G. mellonella* larvae co-infected with *C. albicans* and *S. aureus *. Larvae (*n*=3) were co-infected with *C. albicans* (1×10^5^ larva^−1^) and *S. aureus* (2×10^4^ larva^−1^) for 0, 6, 24 and 48 h at 37 °C. Larvae were embedded, sectioned (10 µm) and visualized using a Cryoviz (Bioinvision, Inc., Cleveland, OH, USA) cryo-imaging system. Point of inoculation, white arrows; nodules, black arrows; extensive melanization, red arrows.

Previous work has visualized the dissemination of *C. albicans* and *S. aureus *, separately, in larvae [39]. In this case cryo-imaging was employed to assess the development of dual infection within *G. mellonella* larvae. Larvae were co-infected with *C. albicans* (1×10^5^ larva^−1^) and *S. aureus* (2×10^4^ larva^−1^) and cryo-imaging was performed at 0, 6, 24 and 48 h post-infection (Fig. 3). Six hours post-infection melanized nodules (black arrows) appeared around the site of inoculation (white arrows) and throughout the host. By 24 h post-infection there was extensive melanization (red arrow) of larval tissue, especially around the site of inoculation and nodules were visible throughout the larva. By 48 h post-infection the survival rate for *G. mellonella* larvae was 10±3.33% and there was extensive melanization throughout the larvae as well as a large number of distinct nodules.

### Analysis of larval immune response to *C. albicans* and *S. aureus* dual infection

**Fig. 4.**
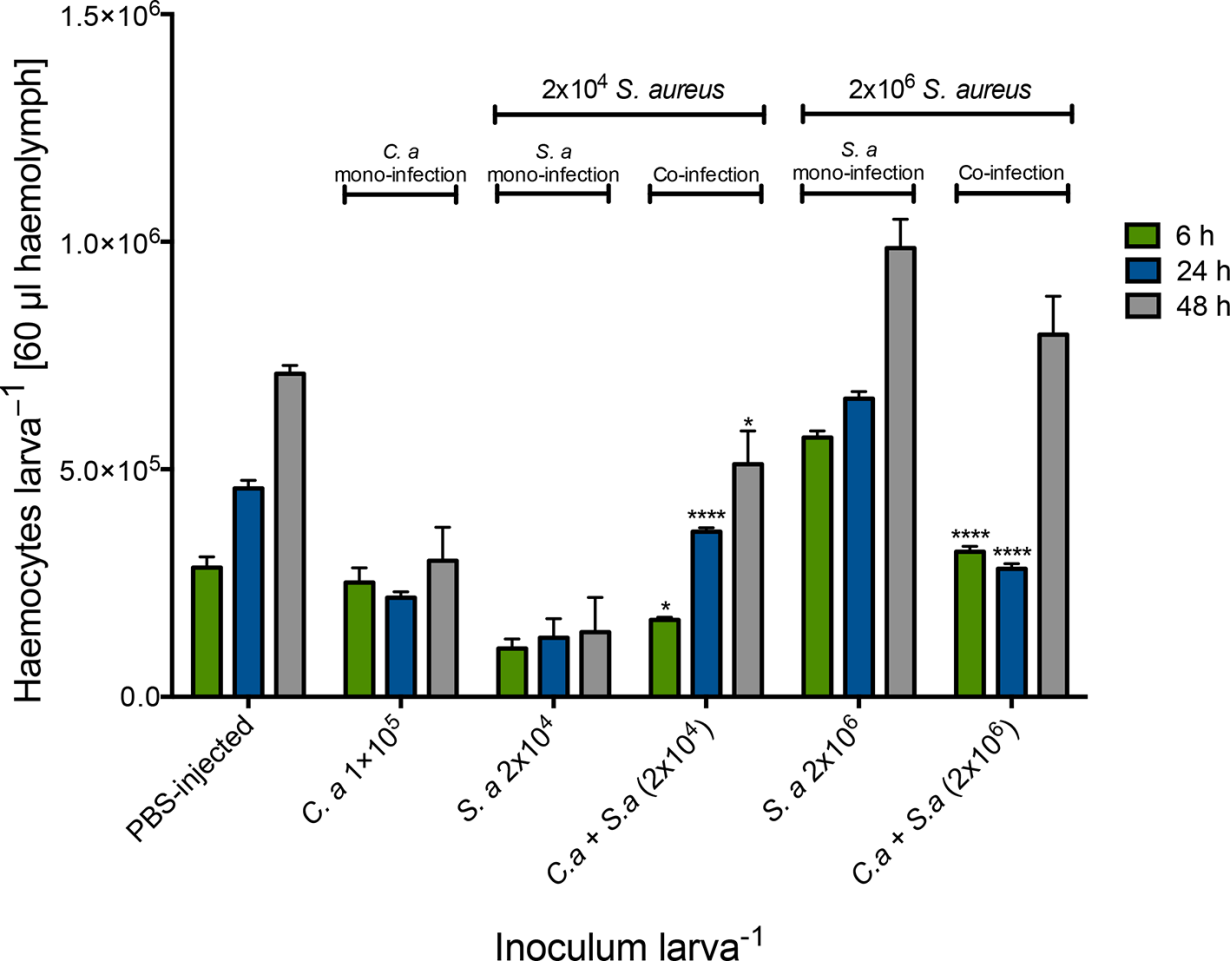
Alteration in circulating haemocyte density following inoculation with *S. aureus* cells alone and in combination with *C. albicans. G. mellonella* larvae were inoculated with 20 µl S. *aureus* cells (2×10^4^ and 2×10^6^ larva^−1^) alone or in combination with *C. albicans* (1×10^5^ larva^−1^) and haemocytes were extracted and enumerated from 6, 24 and 48 h post-inoculation at 37 °C. Statistical analysis was performed by comparing co-infected larvae (*C. a+S.a*) to *S. aureus *-only-treated larvae at their at respective doses and time points (*, *P*<0.05; **, *P*<0.01; ***, *P*<0.001; ****, *P*<0.0001). All values are the mean±se of three independent experiments.

Alterations in circulating haemocyte density in co-infected larvae were determined in order to assess the cellular response to co-infection. Uninfected larvae had a haemocyte density of 2.4±0.17×10^5^ haemocytes larva^−1^. Mono-infection with *C. albicans* (1×10^5^ larva^−1^) resulted in a small alteration in haemocyte density at 24 [2.18±0.22×10^5^ haemocytes larva^−1^ (60 μl haemolymph)] and 48 h (2.09±0.13×10^5^). Inoculation of larvae with *S. aureus* (2×10^4^ larva^−1^) also resulted in a change in haemocyte density at 6 (1.26±0.36×10^5^), 24 (1.30±0.73×10^5^) and 48 h (1.42±0.13×10^5^) post-infection. Mono-infection with *S. aureus* 2×10^6^ larva^−1^ produced alterations in circulating haemocyte density at 6 (5.70±0.25×10^5^, *P*<0.0001) and 24 h (6.55±0.27×10^5^, *P*<0.01) post-infection (Fig. 4).

Co-infection of larvae with *C. albicans* (1×10^5^ larva^−1^) and *S. aureus* (2×10^4^ larva^−1^) resulted in an increase in the density of circulating haemocytes at 6 (1.69±0.13×10^5^, *P*<0.05), 24 (3.63±0.14×10^5^, *P*<0.0001) and 48 h (5.11±0.20×10^5^, *P*<0.05) post-infection compared to larvae infected solely with *S. aureus* (2×10^4^ larva^−1^). Co-infection of *C. albicans* (1×10^5^ larva^−1^) with *S. aureus* at an inoculum of 2×10^6^ larva^−1^ (3.19±0.31×10^5^, *P*<0001) resulted in a significant decrease in circulating haemocyte density relative to larvae infected solely with *S. aureus* (2×10^6^ larva^−1^) 6 h post-infection (2×10^5^, 3.15±0.17×10^5^; 2×10^6^, 5.70±0.25×10^5^).

Quantitative proteomic analysis was performed on the cell-free haemolymph of *G. mellonella* larvae following co-infection with *C. albicans* (1×10^5^ larva^−1^) and *S. aureus* (2×10^4^ larva^−1^) for 0, 6 and 24 h post-infection. In total, 2293 peptides were identified, representing 351 proteins with 2 or more peptides and 48 and 83 (6 vs 0 h and 24 vs 0 h, respectively) proteins were determined to be differentially abundant (ANOVA, *P*<0.05) with a fold change of >1.5. A total of 34, 46 and 84 proteins at 0, 6 and 24 h, respectively, were deemed exclusive (Fig. S1, available in the online version of this article). These proteins were subsequently used to statistically analyse the total differentially expressed group after imputation of the zero values as described and were then included in statistical analysis after data imputation. A principal component analysis (PCA) was performed on all filtered proteins and distinguished the 0, 6 and 24 h *C*. *albicans-* and *S. aureus *-treated samples. A clear difference between the 0, 6 and 24 h proteome was observed (Fig. S2).

**Fig. 5.**
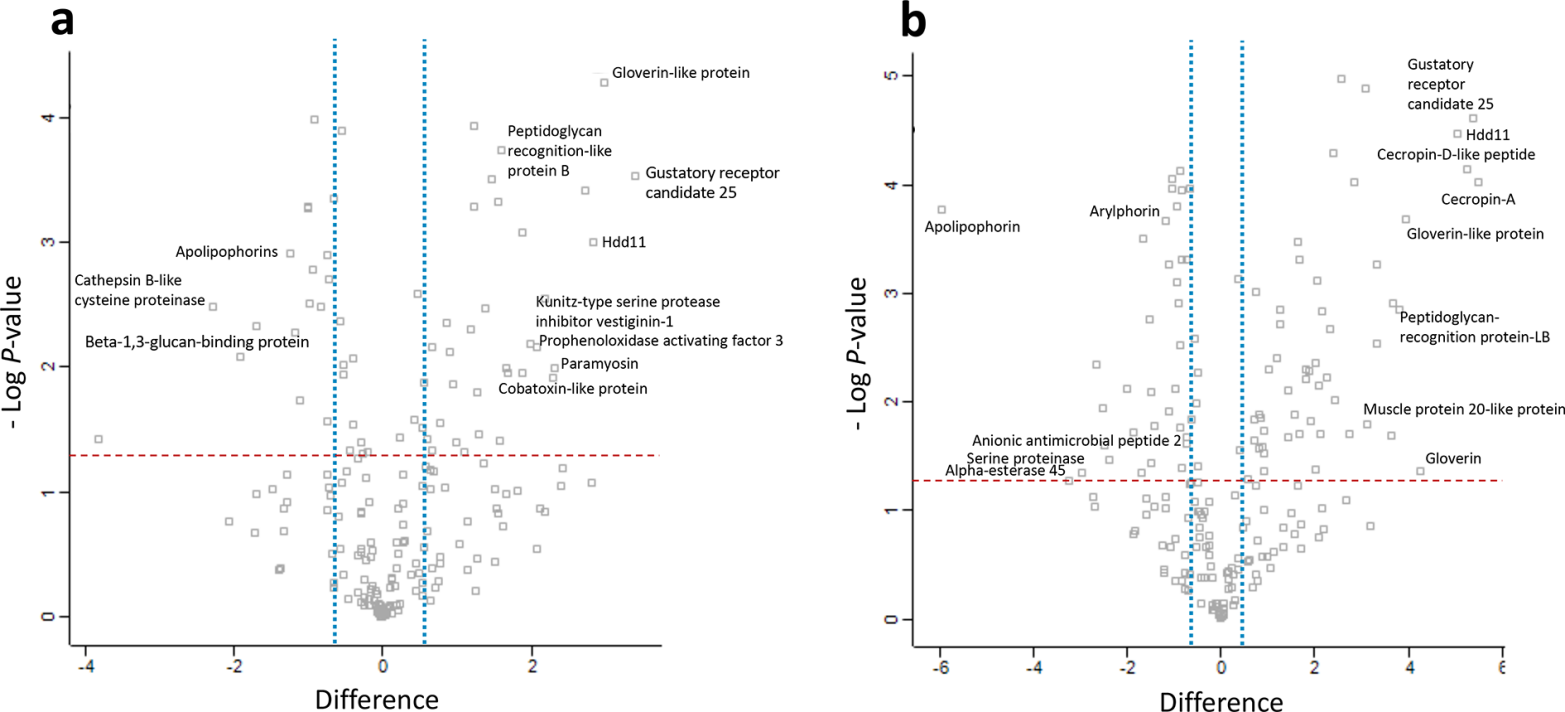
Proteomic responses of *G. mellonella* larval haemolymph following co-infection by *C. albicans* (1×10^5^ larva^−1^) and *S. aureus* (2×10^4^ larva^−1^) after 6 (a) and 24 (b) h at 37 °C. Volcano plots represent protein intensity difference (log_2_ mean intensity difference) and significance in differences (- log P-value) based on a two-sided *t* -test. Proteins above the line are considered statistically significant (*P* value<0.05) and those to the right and left of the vertical lines indicate relative fold changes>1.5. Annotations are given for the most differentially abundant proteins identified in haemolymph from co-infected larvae 6 and 24 h. These plots are based upon post imputed data.

The proteins that showed increased relative abundance in the larvae co-infected for 6 h compared to the control were gustatory receptor candidate 25 (+10.5-fold), gloverin-like protein (+7.9-fold), putative defence protein Hdd11 (+7.1-fold), actin 3 (+6.6-fold) and paramyosin (+5-fold). The proteins that showed decreased relative abundance in the larvae co-infected for 6 h compared to the control were cathepsin B-like cysteine proteinase (−4.9-fold), apolipophorins (−2.4-fold), beta-1, 3-glucan-binding protein (−2.3-fold) and methionine-rich storage protein (−2.2-fold) (Fig. 5a **,** Table S1).

The proteins that showed increased relative abundance in co-infected larvae at 24 h compared to the control larvae were cecropin-A (+45.4-fold), gustatory receptor candidate 25 (+41.7-fold), cecropin-d-like peptide (+37.8-fold), putative defence protein Hdd11 (+33.3-fold), gloverin (+19.3-fold) peptidoglycan-recognition protein-LB (+14.0-fold), serpin-4B (+12.9-fold) and salivary cysteine-rich peptide precursor (+12.6-fold). The proteins that showed decreased relative abundance in larvae co-infected for 24 h compared to the control larvae were apolipophorins (−62.4-fold), alpha-esterase 45 (−7.7-fold), serine proteinase (−6.2-fold), saposin-related precursor (−5.8-fold), carboxylesterase CarE-11 precursor (−5.6-fold) and cathepsin B-like cysteine proteinase (−4-fold) (Fig. 5b **,** Table S2). A range of antimicrobial peptides and proteins (Fig. S3a)**,** proteins associated with bacterial and fungal recognition, and proteins involved in nodule formation (Fig. S3b) were identified within the co-infected larval haemolymph.

A range of *C. albicans* and *S. aureus* proteins were also detected in co-infected *G. mellonella* larval haemolymph. A total of 23 *C*. *albicans* proteins were identified in co-infected *G. mellonella* larval haemolymph and included Hsp70 family ATPase, heat shock protein SSA2 and peroxiredoxin TSA1-A (Table S3). In addition, 20 *S *. *aureus* proteins such as staphopain B and HTH-type transcriptional regulator SarR were detected in haemolymph 24 h post-infection (Table S4).

## Discussion

The incidence of polymicrobial infection is possibly underestimated as current routine clinical microbiological investigation could never encompass the vast number and diversity of microbes present within the human micro- and mycobiome. Advances in technologies associated with the detection of these microbes will eventually enter the clinic and this will require the reclassification of many mono-infections as polymicrobial infections. The work presented here aimed to develop an *in vivo* system to study the pathogenesis of polymicrobial infection using *G. mellonella* larvae, which have been widely used for studying the development of single-species infections [46–49].

Inoculation of larvae with *C. albicans* (1×10^5^ larva^−1^) or *S. aureus* (2×10^4^ larva^−1^) resulted in no reduction in survival, but co-inoculation of larvae with these doses resulted in a large decrease in survival. This suggests that the interactions between *C. albicans* and *S. aureus* may be synergistic, which has previously been documented in murine studies [1, 8, 9, 13, 14, 16, 50]. Infection of *G. mellonella* larvae with *S. aureus* (2×10^6^ larva^−1^) or *C. albicans* (5×10^5^ larva^−1^) in mono- infection resulted in 70 and 44 % larval mortality after 48 h, respectively [34, 38]. Co-infection of larvae with *S. aureus* (1×10^5^ larva^−1^) and *C. albicans* (2×10^4^ larva^−1^) produced mortality of 90 %, 48 h post-infection. Interestingly, single doses of *C. albicans* and *S. aureus* were non-lethal 5 days post-infection in mice but when combined resulted in 70 % mortality 2 days post-infection [50].

Co-inoculation of larvae resulted in a significant increase in *S. aureus* c.f.u larva^−1^ relative to mono-infection. In co-infected mice, the microbial burden was significantly higher in the kidney and spleen compared to mono-infection [50]. Interestingly, the total bacterial c.f.u. in co-infected larvae, regardless of dose, was 10.65±4.40-fold higher than in *S. aureus *-only-infected larvae.

Cryo-imaging was utilized to assess the dissemination of co-infection throughout the host over 48 h. By 24 h there was significant nodule formation and by 48 h there was extensive melanization and widespread nodule formation. Melanization of co-infected larvae is more systemic than that observed in mono-infection, which appears as localized nodules throughout the host [34, 39]. Previous results indicated that infection of larvae with a higher inoculum of *C. albicans* (5×10^5^ larva^−1^) or *S. aureus* (2×10^6^ larva^−1^) in mono-infection resulted in more cuticular melanization and larger granuloma (nodule)-type structure formation [34, 39]. This may indicate that co-infection, at a lower cell density, which ultimately produced a larger decrease in survival relative to mono-infection, may have activated a less robust immune response to co-infection (summarized in Table 1) and via less cuticular melanization. This aberrant immune response (e.g. decreased AMP abundance in co-infection relative to mono-infection) could be due to the acute and accelerated proliferation of *S. aureus* in the presence of *C. albicans*, which results in an uncontrollable bacterial burden that cannot be cleared in time by the host. Peters and Noverr [50] demonstrated that co-infection was associated with an increased inflammatory response characterized by an up to 100-fold increase in inflammatory cytokines (e.g. IL-6) and neutrophil influx [50].

**Table 1.** Comparison of the different proteomic responses between single infection of G. *mellonella* larvae with *C. albicans* [30], *S. aureus* [29] and co-infection (current study). Fold change is 24 h infected larval haemolymph relative to 0 h relevant infected control*

	Infection and reference	*C. albicans* [30]	* S. aureus* [29]	Co-infection (*C. albicans* and *S. aureus *)
	Inoculum size	5×10^5^ 20 µl^−1^	2×10^6^ 20 µl^−1^	1×10^5^ (*C. albicans*) and 2×10^6^ (* S. aureus *) 20 µl^−1^
	Protein name	Fold change (+/−)*	Fold change (+/−)*	Fold change (+/−)*
**Invasion**	Muscle protein 20-like protein	+173.3-fold	nd	+8.6-fold
**Nodulation**	Hdd11	+49.4-fold	+7.24-fold	+33.3-fold
**Antimicrobial peptide**	Gloverin	+52.5-fold	+121.69-fold	+19.3-fold
	Cecropin-D	+22.8-fold	+73.7-fold	+37.8-fold
**Detoxification**	Glutathione s transferase	+114.1-fold	nd	nd
	Thioredoxin	+3.2-fold	−3.94-fold	nd
**Microbial recognition**	PG-RP LB	+71.6-fold	+9.53-fold	+14-fold
	PG-RP B	+7.5-fold	+17.35-fold	nd
	β-glucan RP	−28.6-fold	+2.16-fold	nd

nd, non detected; PG RP, peptidoglycan recognition protein.

Infection of larvae with a low inoculum of *S. aureus* (2×10^4^ larva^−1^) with *C. albicans* (1×10^5^ larva^−1^) resulted in a significant increase in haemocyte density, however an inoculum of *S. aureus* (2×10^6^ larva^−1^) with *C. albicans* (1×10^5^ larva^−1^) produced a decrease in haemocyte density compared to *S. aureus* mono-infection. The increase in haemocyte density is probably a result of the release of haemocytes usually attached to the linings of internal surface of the cuticle and organs such as the fat body [51], while the decrease in haemocyte density associated with the higher inoculum may be due to the aggregation of haemocytes at the site of inoculation in order to halt the spread of the infection.

The humoral immune response of larvae to co-infection was determined by label-free MS. At 6 h post co-infection antimicrobial peptide gloverin-like protein had increased 7.9-fold. Gloverins are heat-stable antibacterial polypeptides that bind to bacterial lipopolysaccharide and components of the fungal cell wall. It was previously demonstrated that *Escherichia coli* and *S. aureus* induce gloverin expression in *Bombyx mori* and *Manduca sexta* [52, 53]. Gloverins display activity against yeasts but do not possess anti-* S. aureus* activity [53]. In *C. albicans*-infected *G. mellonella* larvae, gloverin was not detected 6 h post-infection but had increased 52-fold at 24 h post-infection, however in *S. aureus *-infected larvae gloverin had increased 20-fold at 6 h and 121-fold at 24 h post-infection [34, 38]. Similarly, PGRP-B increased 3-fold in co-infected larvae but 2.3-fold in *C. albicans* and 6-fold in *S. aureus* mono-infected larvae [34, 38]. These experiments were performed with five times more *C. albicans* (5×10^5^ larva^−1^) and 100 times more *S. aureus* (2×10^6^ larva^−1^) compared to co-infected larvae [*C. albicans* (1×10^5^ larva^−1^), *S. aureus* (2×10^4^ larva^−1^)] in the current study (Table 1). Members of the phenoloxidase cascade (e.g. prophenoloxidase activating factor 3) increased in abundance by 4.2-fold in co-infected larvae 6 h post-co-infection but had only increased 2.4-fold in *C. albicans* and 2.85-fold in *S. aureus* at 6 h post-mono-infection (Table 1). The phenoloxidase cascade is a series of enzymatic reactions that result in the formation of phenoloxidase, which participates in melanotic encapsulation, wound healing and cuticle sclerotization, and this reaction is analogous to the mammalian complement cascade in terms of protein structure and function [54].

By 24 h post-co-infection, the abundance of cecropin A had increased 45-fold as compared to control larvae. Cecropin A is a 37 amino acid antimicrobial peptide first isolated from *Hyalophora cecropia*. It demonstrates antibacterial activity against multidrug-resistant bacteria (e.g. *S. aureus *), induces *C. albicans* apoptosis and has immune-modulatory effects on macrophages [55–57]. Cecropin A had increased in *C. albicans* mono-infected larvae (4.5-fold) at 6 h post-infection, but increased 10.5-fold 24 h post-* S. aureus* infection [34, 38]. Moricin-like peptide increased 21- and 20.8-fold during *C. albicans* and *S. aureus* mono-infection, respectively, but was not detected in co-infected larvae. Moricins are secreted pro-peptides that are activated via proteolysis and increase the permeability of bacterial and fungal membranes. *G. mellonella* moricins are highly active against yeasts and *S. aureus* [58, 59]. Hdd11 had increased by 7.1- and 33.33-fold at 6 and 24 h post-co-infection. However, Hdd11, which shares homology with noduler from the tasar silkworm (*Antheraea mylitta*) [60, 61]*,* had increased by 49-fold and 7.24-fold at 24 h post*-C. albicans* and *S. aureus* infection, respectively. Noduler shares a reeler domain with Hdd11 and binds insect haemocytes, bacterial LPS and fungal beta glucan, is enriched within nodules (*G. mellonella* nodules are highly enriched for Hdd11 protein; unpublished observation) and is important during the nodulation response [62]. Apolipophorin decreased 1.9-fold in abundance in *C. albicans* mono-infected larvae and was not detected during *S. aureus* infection, but was found to be significantly decreased (64.2-fold) during co-infection by *S. aureus* and *C. albicans*. Apolipophorin plays a role in lipid transport [63, 64] but has immune-modulatory activity and augments the activity of lysozyme [65], potentiates the activity of antimicrobial peptides [66], regulates phenoloxidase activity [67, 68] and is a pathogen recognition receptor and opsonin of lipopolysaccharide, lipoteichoic acids and fungal β-glucan [69]. Furthermore, lysozyme decreased (4.9-fold) during *C. albicans* mono-infection of larvae and increased (31.38-fold) during *S. aureus* mono-infection, but was not detected in co-infected larvae.

It is possible that co-infection with *C. albicans and S. aureus* activates a less robust immune response in terms of recognition and opsonin proteins (PGRPs), which are essential for early microbial recognition to prevent the spread of infection. Interestingly, the fungicidal AMP gloverin decreased in abundance during co-infection relative to mono-infection by both organisms, which may indicate a defective killing response of larvae to *C. albicans*, which, in turn, may allow increased proliferation of *S. aureus* due to increased survival of *C. albicans*.

In total, 23 *C*. *albicans* and 20 *S *. *aureus* proteins were found in haemolymph during co-infection of *G. mellonella* larvae. *C. albicans* heat shock protein SSA2 and peroxiredoxin TSA1-A and *S. aureus* staphopain B were found in haemolymph 24 h post-infection. SSA1 can induce host cell endocytosis of *C. albicans*, which leads to increased virulence [70] and plays a role in resistance to antimicrobial peptides and antifungal agents [71–73]. Peroxiredoxin TSA1-A was also detected in larvae mono-infected with *C. albicans* [39] and plays a role in cell protection against oxidative stress [74]. *S. aureus* staphopain B decreased the phagocytic activity of neutrophils and cleaved CD31, resulting in increased clearance by macrophages [75]. It is possible that these proteins display activity against insect haemocytes and antimicrobial peptides or play a role in detoxification of the microbial cell from the host immune response.

Co-infection with *C. albicans* and *S. aureus* produced elevated mortality relative to mono-infection and this is associated with increased *S. aureus* proliferation, alterations in circulating haemocyte densities and systemic dissemination of infection throughout larvae. The immune proteome of co-infected larvae displays an altered immune response relative to infection by *C. albicans* [38] or *S. aureus* [34] at a higher inoculum. This study demonstrates that the *G. mellonella* larvae may be a useful *in vivo* system to study co-infection by dual-species, inter-kingdom microbial pathogens. This model could be adapted for other common interactions (antagonistic or synergistic) that exist during human infection. This is particularly important as advances in healthcare that ultimately result in inhibition of the host immune response (e.g. SYK inhibitors) will facilitate the emergence of new pathogens and co-infection by diverse pathogens that are well adapted to thrive in the immunocompromised host.

## Supplementary Data

Supplementary material 1
