# Supplementary material for: Candida albicans increases the pathogenicity of Staphylococcus aureus during polymicrobial infection of Galleria mellonella larvae
Source: Microbiology (Reading). 2020 Feb 18;166(4):375–85. doi: 10.1099/mic.0.000892 (PMC7377259; doi:10.1099/mic.0.000892)
Supplement: Supplementary material 1 [file mic-166-375-s001.pdf]

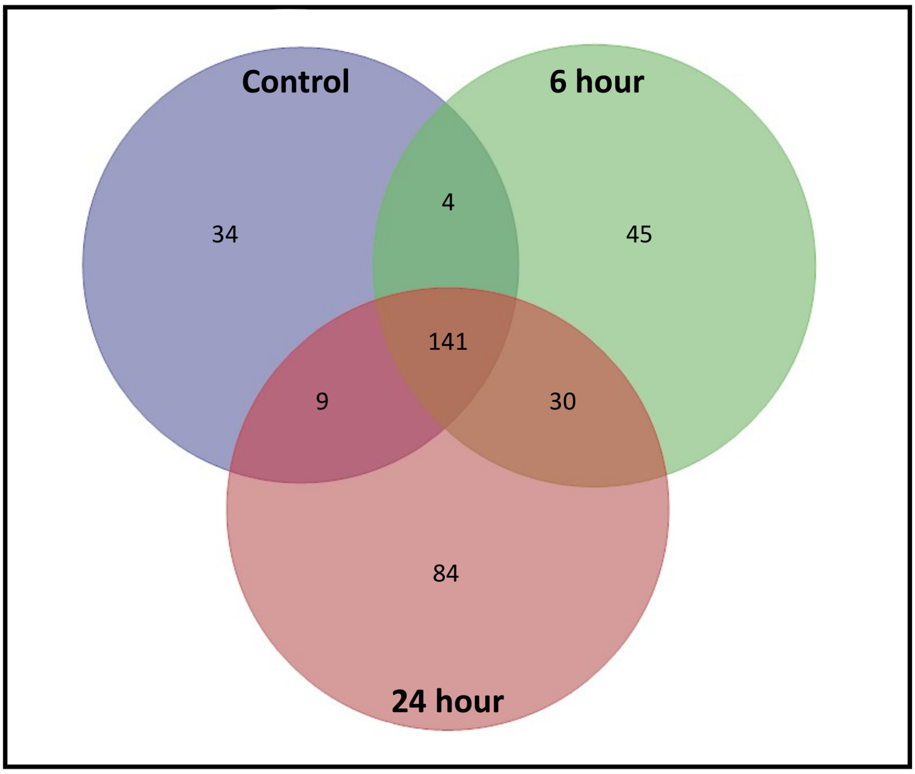

Figure S1. Venn diagram of number of proteins detected in control, 6 h and 24 h co infected larvae. In total 34, 45 and 84 proteins were deemed exclusive to the control, 6 h and 24 h hemolymph proteome from co-infected larvae.

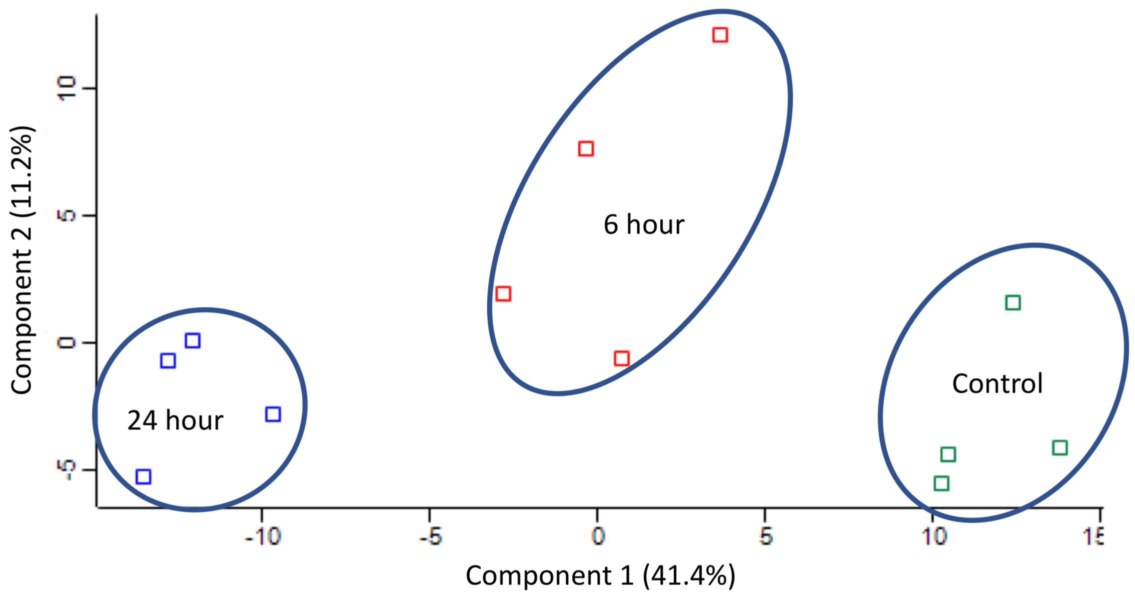

Figure S2. Principal component analysis (PCA) of *G. mellonella* hemolymph proteomic profiles following co-infection with *C. albicans* and *S. aureus* for 0, 6 and 24 h. PCA of three replicates of each treatment included in LFQ analysis with a clear distinction between each time point.

Table S1: Proteins changed (increased [+] and decreased [-]) in abundance in *G. mellonella* larvae co-infected with *C. albicans* [1×10^5 larva^{-1}] *S. aureus* [2×10^4 larva^{-1}]) at 6 h relative to control larvae

Protein name	Number of peptides	Sequence coverage [%]	Score	P-value	Fold Change
gustatory receptor candidate 25	5	34	79.852	0.000299	+10.5
gloverin-like protein, partial	3	16.9	162.16	4.77E-05	+7.9
Putative defense protein Hdd11	8	60.7	131.58	0.001001	+7.1
actin 3	8	30.9	148.38	0.000381	+6.6
paramyosin, putative	5	21.3	44.51	0.010233	+5.0
odorant-binding protein	3	8.9	24.256	0.01226	+4.9
integument esterase 2 precursor	7	33.6	54.189	0.002835	+4.6
prophenoloxidase activating factor 3	8	45.5	99.646	0.006993	+4.2
Kunitz-type serine protease inhibitor vestiginin-1	3	17	23.033	0.00659	+4.0
putative protease inhibitor 4	7	24.3	239.81	0.00084	+3.7
serine protease inhibitor 5 precursor	6	17.2	58.799	0.01111	+3.6
cobatoxin-like protein	2	15.8	63.982	0.011142	+3.2
serine proteinase-like protein 2	3	40.2	52.672	0.010279	+3.1
peptidoglycan recognition-like protein B, partial	13	34	135.36	0.000185	+3.0
thymosin isoform 1	5	42.7	64.28	0.038421	+3.0
Serine protease inhibitor dipetalogastin	14	28.8	323.31	0.000475	+2.9
nimrod B precursor	2	21.7	134.44	0.000309	+2.7
GI17397	4	53.2	92.275	0.003409	+2.6
conserved hypothetical protein	4	22.6	33.183	0.034018	+2.5
beta actin	12	54.1	323.31	0.015716	+2.4
kazal-type proteinase inhibitor precursor	6	21.4	155.94	0.000117	+2.4
paramyosin, putative	19	43	323.31	0.000521	+2.3
cellular retinoic acid binding protein	15	35.6	179.65	0.004901	+2.3
prophenol oxidase activating enzyme 3	16	41.6	171.68	0.047205	+2.2
chemosensory protein	4	24.8	35.034	0.039315	+2.0
spodoptericin, partial	4	33.5	145.34	0.013664	+1.9
cationic peptide CP8 precursor	8	29.2	323.31	0.007608	+1.9
diapause bioclock protein	4	62.6	234.71	0.004356	+1.8
chemosensory protein	5	16.7	63.037	0.027691	+1.7
Inducible serine protease inhibitor 2	4	13.2	112.66	0.006884	+1.6
AGAP011516-PA, partial	7	44.4	79.314	0.046237	+1.6
Gelsolin	24	36.9	323.31	0.037978	-1.5
juvenile hormone binding protein	15	44.5	323.31	0.000451	-1.6
arylphorin	81	78.6	323.31	0.001997	-1.7
uncharacterized protein Dmel_CG33290	2	11.9	33.278	0.027253	-1.7
arylphorin	55	79.8	323.31	0.001283	-1.7
hypothetical protein	16	37.1	323.31	0.003286	-1.8

Apolipophorins	9	53	254.29	0.000104	-1.9
adhesion related protein, transmembrane (plasmid)	9	37.2	129.75	0.001665	-1.9
unknown [Picea sitchensis]	11	29.8	216.69	0.003057	-2.0
hypothetical protein, partial	5	13.3	81.426	0.000517	-2.0
beta-1,3-glucan recognition protein precursor	17	32.3	175.69	0.000531	-2.0
methionine-rich storage protein	18	81.4	323.31	0.018393	-2.2
Beta-1,3-glucan-binding protein	2	14.5	53.396	0.005218	-2.3
Apolipophorins	1	13.8	18.495	0.001219	-2.4
unknown [Picea sitchensis]	5	13.2	45.134	0.00831	-3.8
cathepsin B-like cysteine proteinase	7	24.3	60.827	0.003242	-4.9
unknown [Picea sitchensis]	3	23.3	42.505	0.03811	-14.2

Table S2: Proteins changed (increased [+] and decreased [-]) in abundance in *G. mellonella* larvae co-infected with *C. albicans* [1×10^5 larva^{-1}] *S. aureus* [2×10^4 larva^{-1}]) at 24 h relative to control larvae

**Protein name**	**Number of peptides**	**Sequence coverage [%]**	**Score**	**P-value**	**Fold change**
Cecropin-A	2	12.7	18.93	9.31E-05	+45.4
gustatory receptor candidate 25	5	34	79.852	2.5E-05	+41.7
Cecropin-D-like peptide	2	14.5	45.092	7.27E-05	+37.8
Putative defense protein Hdd11	8	60.7	131.58	3.34E-05	+33.3
gloverin	4	59.6	36.145	0.043792	+19.3
gloverin-like protein	3	16.9	162.16	0.000207	+15.3
peptidoglycan-recognition protein-LB	3	72.4	31.878	0.001428	+14.0
serpin-4B	5	78.8	51.837	0.001239	+12.9
salivary cysteine-rich peptide precursor	3	13.5	37.275	0.020182	+12.6
hypothetical protein TTHERM_00163900	4	21.7	40.212	0.002905	+10.1
DNA-directed RNA polymerase II subunit RPB1	3	15.5	59.002	0.000532	+10.0
muscle protein 20-like protein	7	32.5	184.74	0.016145	+8.6
peptidoglycan recognition-like protein B	13	34	135.36	1.28E-05	+8.5
Inducible metalloproteinase inhibitor protein	7	33.2	78.171	9.47E-05	+7.1
hypothetical protein (macronuclear)	7	31	100.56	0.019526	+6.8
protease inhibitor 1 precursor	3	21.2	175.06	1.04E-05	+6.0
heat shock protein hsp21.4	8	18.6	94.178	0.00975	+5.4
Serine protease inhibitor dipetalogastin	14	28.8	323.31	5.01E-05	+5.3
cobatoxin-like protein	2	15.8	63.982	0.002108	+5.0
prophenoloxidase activating factor 3	8	45.5	99.646	0.005944	+4.8
actin 3	8	30.9	148.38	0.001451	+4.5
paramyosin, putative	5	21.3	44.51	0.019479	+4.3
chemosensory protein 7 precursor	8	30.8	124.29	0.006951	+4.3

beta actin	12	54.1	323.31	0.000762	+4.1
serpin 3a	9	13.2	122.9	0.041815	+4.1
integument esterase 2 precursor	7	33.6	54.189	0.004394	+4.0
Kunitz-type serine protease inhibitor vestiginin-1	3	17	23.033	0.014824	+3.8
hemolin, partial	17	66	306.28	0.005263	+3.7
prophenol oxidase activating enzyme 3	16	41.6	171.68	0.006246	+3.5
chemosensory protein	4	24.8	35.034	0.005067	+3.5
thymosin isoform 1	5	42.7	64.28	0.019699	+3.2
peptidoglycan recognition protein precursor	12	53	272.37	0.000495	+3.2
hemolin	7	56.4	228.83	0.000336	+3.1
putative protease inhibitor 4	7	24.3	239.81	0.012987	+3.0
serine proteinase-like protein 2	3	40.2	52.672	0.021263	+2.7
AGAP011516-PA, partial	7	44.4	79.314	0.007866	+2.7
kazal-type proteinase inhibitor precursor	6	21.4	155.94	0.001432	+2.4
nimrod B precursor	2	21.7	134.44	0.001901	+2.4
GI17397	4	53.2	92.275	0.003949	+2.3
myophilin	5	18.9	50.592	0.004987	+2.0
hypothetical protein 29, partial	7	32.2	60.846	0.043305	+1.9
cationic peptide CP8 precursor	8	29.2	323.31	0.018288	+1.9
hypothetical protein	37	67.5	323.31	0.025558	+1.9
paramyosin, putative	19	43	323.31	0.014214	+1.8
serine protease inhibitor 11 precursor	10	23.1	116.7	0.027161	+1.8
Inducible serine protease inhibitor 2	4	13.2	112.66	0.013159	+1.8
unknown, partial [Helicoverpa armigera]	20	36.6	274.94	0.000967	+1.7
diapause bioclock protein	4	62.6	234.71	0.014342	+1.7
Gelsolin	24	36.9	323.31	0.022655	+1.6
uncharacterized protein LOC661483	6	13.9	120.98	0.014646	+1.5
unknown [Picea sitchensis]	21	48.5	323.31	0.00011	-1.6
Anionic antimicrobial peptide 2	10	32.9	312.43	0.023877	-1.7
beta-galactosidase	23	35.2	297.16	0.00048	-1.7
aminoacylase	9	24.5	89.608	0.02118	-1.7
Apolipophorins	12	85.9	323.31	0.00049	-1.8
carboxylesterase clade H, member 1 precursor	25	69.1	323.31	0.000112	-1.8
heat shock protein 25.4 precursor	14	40.5	323.31	0.040029	-1.8
C-type lectin 21 precursor	11	31.2	161.97	0.017307	-1.8
juvenile hormone binding protein	15	44.5	323.31	0.003041	-1.8
apolipophorin, partial	16	47.9	323.31	7.37E-05	-1.8
adhesion related protein, transmembrane (plasmid)	9	37.2	129.75	0.001229	-1.8
putative serine protease-like protein 2	20	71	323.31	0.000156	-1.9
hypothetical protein, partial	5	13.3	81.426	0.000798	-1.9
unknown [Picea sitchensis]	11	29.8	216.69	0.007533	-2.0
similar to CG10638-PA	4	28.8	205.96	0.00011	-2.0

Apolipophorins	9	53	254.29	8.95E-05	-2.1
carboxylesterase clade H, member 1 precursor	10	39.3	209.21	0.012125	-2.1
arylphorin	81	78.6	323.31	0.000548	-2.2
arylphorin	55	79.8	323.31	0.000218	-2.2
juvenile hormone binding protein	17	56.4	323.31	0.016559	-2.6
abnormal wing disc-like protein	10	52.6	120.66	0.00805	-2.8
Fibrohexamerin	14	18	323.31	0.03681	-2.8
methionine-rich storage protein	18	81.4	323.31	0.001717	-2.9
hypothetical protein	16	37.1	323.31	0.000316	-3.1
unknown [Picea sitchensis]	7	20.1	70.525	0.044628	-3.2
unknown [Picea sitchensis]	5	13.2	45.134	0.019255	-3.7
cathepsin B-like cysteine proteinase	7	24.3	60.827	0.007651	-4.0
hypothetical protein, partial	3	7.7	21.784	0.034442	-5.2
carboxylesterase CarE-11 precursor	4	33.1	65.687	0.025218	-5.6
saposin-related precursor	10	31.4	78.529	0.011407	-5.8
serine proteinase	15	35.8	323.31	0.004564	-6.2
alpha-esterase 45	14	52.9	285.29	0.044877	-7.7
Apolipophorin	1	13.8	18.495	0.000172	-62.4

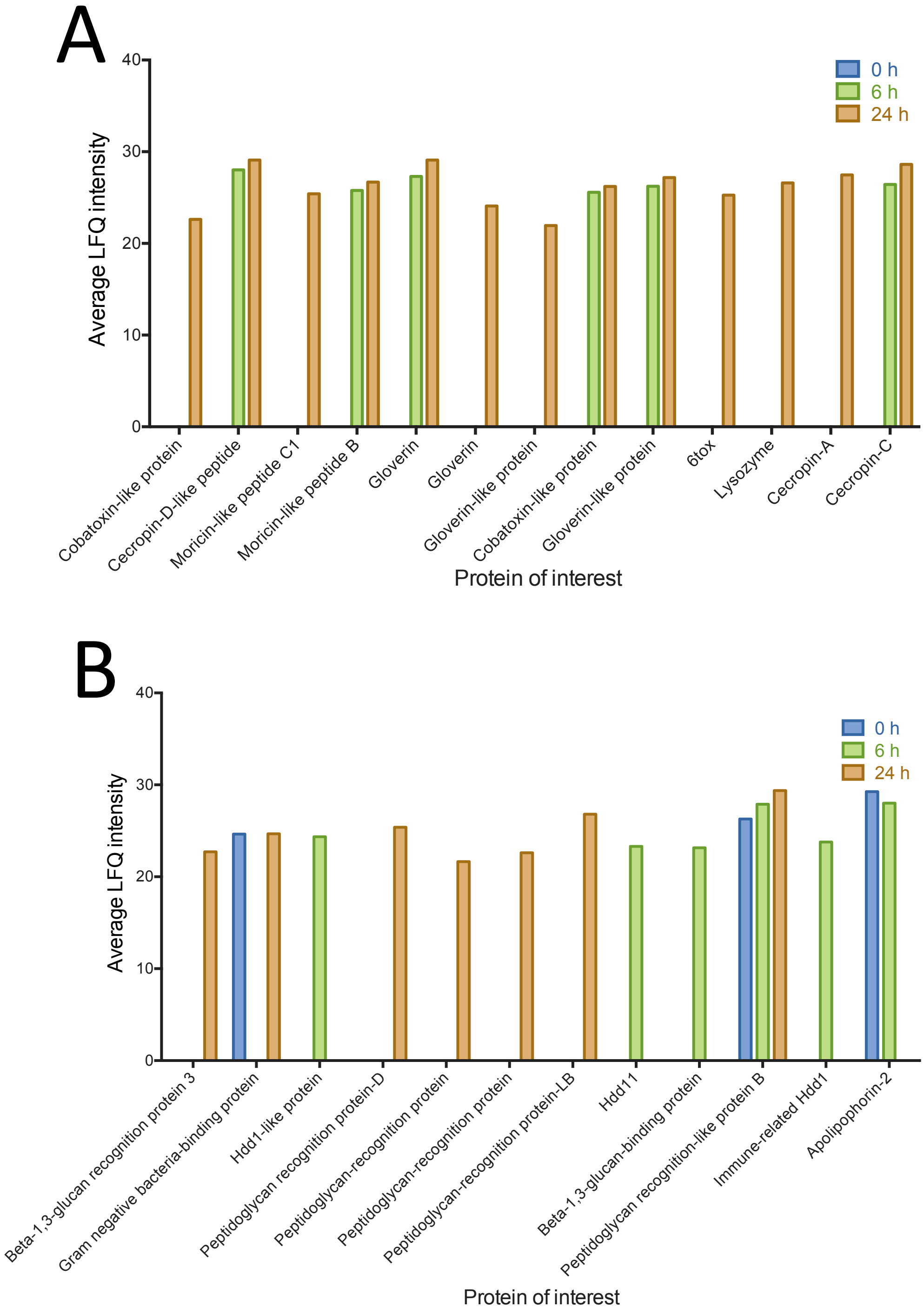

Figure S3. Average LFQ intensities for defence proteins detected in co-infected larval hemolymph. The relative average LFQ intensity values for a range of antimicrobial peptides and proteins (A), proteins involved in bacterial and fungal cell recognition and proteins which play a key role in nodule formation (B) in *G. mellonella* larvae are shown over 0, 6 and 24 h post infection.

Table S3. List of *C. albicans* proteins detected in *G. mellonella* larval hemolymph at 24 hours post co infection with *C. albicans* [1 × 10^5 larva^{-1}] *S. aureus* [2 × 10^4 larva^{-1}]).

Uniprot ID	Protein IDs
**A0A1D8PEV2**	Alkaline phosphatase (EC 3.1.3.1)
**A0A1D8PFM7**	Uncharacterized protein
**A0A1D8PG96**	Hsp70 family ATPase
**A0A1D8PGY6**	Uncharacterized protein
**A0A1D8PS79**	Isocitrate dehydrogenase [NADP] (EC 1.1.1.42)
**A0A1D8PI81**	Ifm3p
**A0A1D8PIA8**	Uncharacterized protein
**A0A1D8PKC4**	Ubiquitin-specific protease
**A0A1D8PKJ3**	E1 ubiquitin-activating protein
**A0A1D8PKJ4**	Saccharopine dehydrogenase (NADP+, L-glutamate-forming)
**A0A1D8PL61**	Midasin
**A0A1D8PRP3**	Mms22p
**A0A1D8PT92**	Uncharacterized protein
**A0A1D8PU73**	Uncharacterized protein
**Q9Y7F0**	Peroxiredoxin TSA1-A (EC 1.11.1.15) (Thiol-specific antioxidant protein) (Thioredoxin)
**Q59QD6**	Elongation factor 1-alpha 2 (EF-1-alpha 2)
**P25997**	Elongation factor 3 (EF-3)
**P46587**	Heat shock protein SSA2
**Q59KI4**	Chromatin-remodeling ATPase INO80 (EC 3.6.4.-)
**Q59LZ4**	Uncharacterized protein
**Q59VP2**	Histone H2A.2
**Q5AAT3**	Uncharacterized protein
**Q5AG31**	Mediator of RNA polymerase II transcription subunit 14 (Mediator complex subunit 14)

Table S4. List of *S. aureus* proteins detected in *G. mellonella* larval hemolymph at 24 hours post co infection with *C. albicans* [1×10^5 larva^{-1}] *S. aureus* [2×10^4 larva^{-1}]).

Uniprot ID	Protein IDs
**P69848**	GTP 3',8-cyclase (EC 4.1.99.22) (Molybdenum cofactor biosynthesis protein A)
**Q2FUW0**	Uncharacterized protein
**Q2FVC2**	Pyrophosphohydrolase, putative
**Q2FVF4**	Glutamate synthase alpha subunit, putative (EC 1.4.1.13)
**Q2FWP8**	Phi PVL orf 32-like protein
**Q2FX03**	UPF0421 protein SAOUHSC_02103
**Q2FX51**	Phage terminase, large subunit, PBSX family
**Q2FX65**	Phage tape measure protein
**Q2FY35**	Probable glycine dehydrogenase (decarboxylating) subunit 2 (EC 1.4.4.2) (Glycine cleavage system P-protein subunit 2) (Glycine decarboxylase subunit 2) (Glycine dehydrogenase (aminomethyl-transferring) subunit 2)
**Q2FZ20**	Polyribonucleotide nucleotidyltransferase (EC 2.7.7.8) (Polynucleotide phosphorylase) (PNPase)
**Q2FZF9**	Glycerophosphoryl diester phosphodiesterase, putative (EC 3.1.4.46)
**Q2FZL3**	Staphopain B (EC 3.4.22.-) (Staphylococcal cysteine proteinase B) (Staphylopain B)
**Q2FZS6**	2-isopropylmalate synthase, putative (EC 2.3.3.13)
**Q2G012**	Extracellular matrix protein-binding protein emp
**Q2G146**	Uncharacterized protein
**Q2G1H6**	Acetylglutamate kinase (EC 2.7.2.8) (N-acetyl-L-glutamate 5-phosphotransferase) (NAG kinase) (NAGK)
**Q2G1S8**	Uncharacterized protein
**Q2G2A7**	Spermidine/putrescine import ATP-binding protein PotA (EC 3.6.3.31)
**Q2G2D3**	Ribosome maturation factor RimP
**Q9F0R1**	HTH-type transcriptional regulator SarR (Staphylococcal accessory regulator R)
